# A Case of Thoraco-Omphalopagus Conjoined Twins: Clinical Imaging and Anatomical Classification

**DOI:** 10.7759/cureus.50452

**Published:** 2023-12-13

**Authors:** Attka Maryam, Sara Khan, Tarek Alambrouk, Haider Hilal

**Affiliations:** 1 Department of Radiology, Rashid Latif Medical University, Lahore, PAK; 2 Department of Anatomy, St. George's University, Newcastle, GBR; 3 School of Medicine, St. George's University, Newcastle, GBR

**Keywords:** thoraco-omphalopagus, conjoined twins, congenital, development, anatomical classification

## Abstract

Conjoined twins are a rare phenomenon estimated to occur in a range between 1 in 49,000 births and 1 in 189,000 births. As a product of monochorionic-monoamniotic pregnancies, they are currently believed to result from late, incomplete fission of the bilaminar embryonic disk at 13-15 days gestation. Conjoined twins are typically classified by the point at which their bodies are joined, with 15 recognized types, five of which account for more than 70% of cases. Fusion of the thorax and upper abdomen (thoraco-omphalopagus) accounts for 28% of all cases. Mortality and morbidity rates remain high irrespective of the point of fusion, with 40-60% of cases being lost to miscarriage and stillbirth, and only about 18% of live births surviving more than 24 hours. Given this prognosis, knowledge of underlying anatomy and clinical imaging is paramount to antenatal diagnosis, assessment of viability, and subsequent management of conjoined twins. A case of thoraco-omphalopagus twins with a single heart and single liver discovered on routine ultrasound at 12 weeks gestation is described.

## Introduction

Conjoined twins are a rare phenomenon, with an estimated occurrence ranging between 1 in 49,000 births and 1 in 189,000 births. This rarity, coupled with their obvious divergence from anatomical norms, means that they have been described and documented very diligently [1,2]. Despite this universal fascination with conjoined twins throughout history, the first successful surgical separation of conjoined twins was not achieved until 1955. Furthermore, the etiology of the condition is still not fully comprehended, underscoring the complexity of the subject and the need for continued research and exploration in this field.

As an exclusive product of monochorionic-monoamniotic pregnancies, the prevailing belief is that they result from late, incomplete fission of the bilaminar embryonic disk at 13-15 days gestation. The competing fission theory, which postulates that conjoined twins are derived from the early fusion of two separate monoamniotic twins, is not widely accepted within the scientific understanding. Instead, the incomplete separation hypothesis remains the primary explanation for the causation of conjoined twins.

This review was previously presented as a meeting abstract at the 2018 BACA Winter Scientific Meeting on December 13, 2018.

## Case presentation

A 27-year-old female presented to the Outpatient Department at Arif Memorial Teaching Hospital for obstetric ultrasound. The purpose of this ultrasound was to verify a prior diagnosis of a twin pregnancy. It's worth noting that previous assessments had been conducted at a basic primary healthcare center. This case report was reported in accordance with ethical guidelines and obtained approval from Rashid Latif Medical University (approval number: IRB/2018/004). The patient provided informed consent by signing a waiver and agreeing to the collection of history and images for the case report.

The patient was from a low-income household with a background of consanguineous marriage. There was no family history of multi-fetal pregnancies or any history of congenital anomalies in any first-degree relatives. Her previous pregnancy had resulted in the natural delivery of a male child, who was now three years old, with no significant health or developmental concerns.

**Figure 1:**
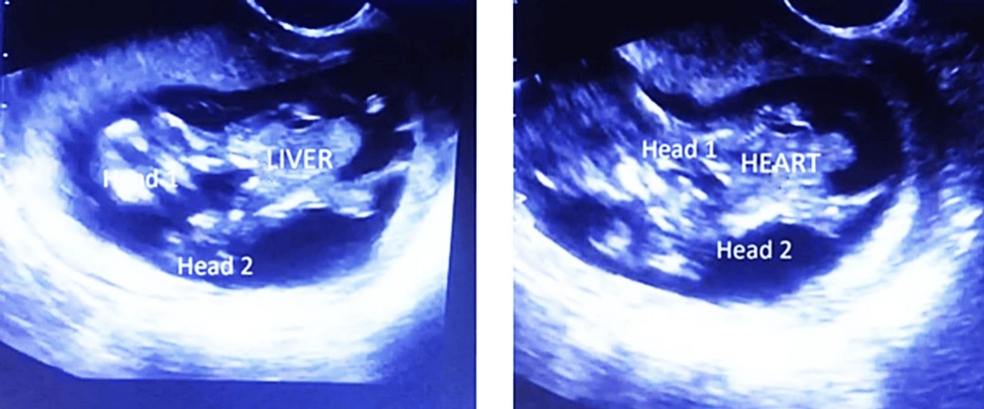
Ultrasound images showing the conjoined fetus

Transabdominal ultrasound was performed with an Edan U2 diagnostic ultrasound machine (EDAN Instruments, Inc., Shenzhen, China) using a curvilinear probe of 7.5 MHz. Two male twins, estimated at 12+ 1 week’s gestation, were noted. Each twin had one head, two arms and legs, and a spine. The fetuses were noted to be fused at their thorax and upper abdomen, as shown in Figure 1. A common heart was noted in the midline. A single liver was observed on the median plane.

**Figure 2:**
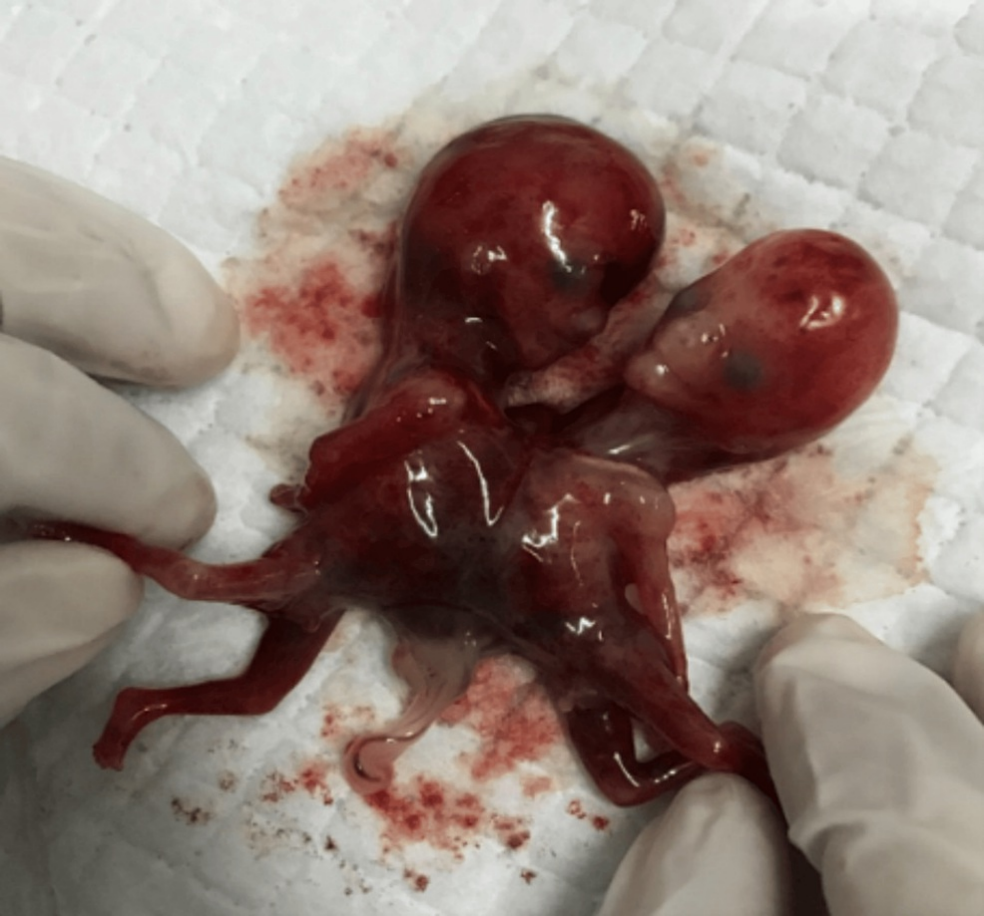
Gross image of the conjoined fetus

On the basis of these anatomical findings, a diagnosis of thoraco-omphalopagus conjoined twins was made. The presence of two shared vital organs, in particular the single heart, essentially excluded the possibility of successful separation surgery and indicated a low probability of survival. The patient was informed of the findings and prognosis. The patient then opted to terminate the pregnancy. A medical abortion was carried out. Examination of the aborted fetus confirmed the ultrasonographic finding, as shown in Figure 2.

## Discussion

**Figure 3:**
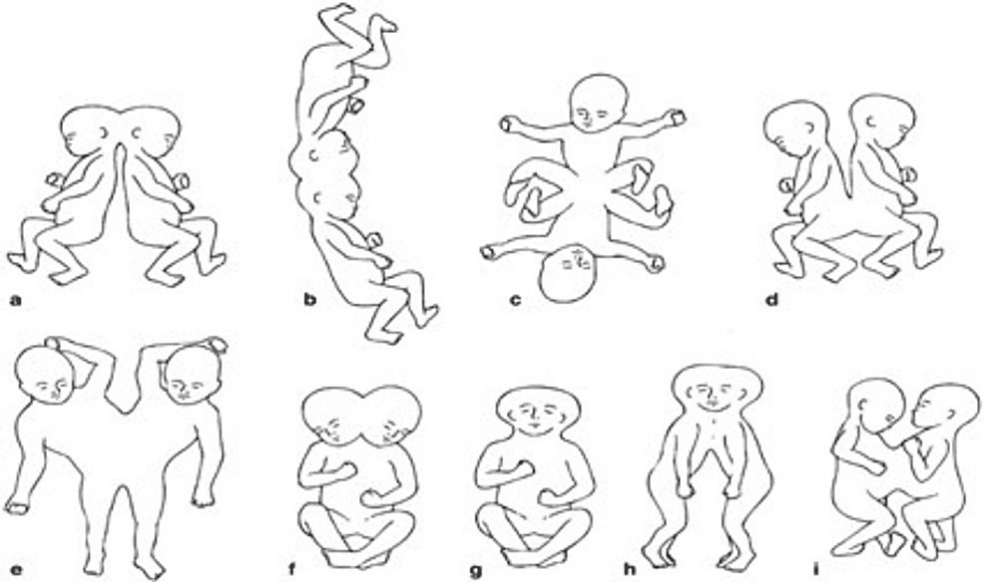
Classification of conjoined twins: (a) craniopagus-occipital fusion, (b) craniopagus-parietal fusion, (c) ischiopagus, (d) pygopagus, (e) parapagus, (f) dicephalus, (g) syncephalus, (h) cephalothoracopagus, (i) thoracopagus Adapted from "The embryology of conjoined twins" by M.H Kaufman, Child's Nervous system, Copyright 2004 by M.H Kaufman. Adapted with permission.

The earliest depictions of conjoined twins date back to the Neolithic period, with further depictions in early Peruvian ceramics dating to 300 CE. Further references and depictions are found throughout historical literature from the Middle East, Europe, and Asia [1,2]. Conjoined twins are typically classified based on the specific point at which their bodies are joined. There are a total of 15 recognized types, with five of these types accounting for more than 70% of all cases. Among these, a fusion of the thorax and upper abdomen (thoraco-omphalopagus) is the most common, comprising 28% of all cases. The principal patterns of fusion are illustrated below in Figure 3 [2,3].

Mortality and morbidity rates for conjoined twins remain high, irrespective of the specific point of fusion. A significant proportion of cases, 40-60%, are lost to miscarriage and stillbirth, and approximately only 18% of live births survive beyond the first 24 hours. Given this prognosis, knowledge of underlying anatomy and clinical imaging is paramount to antenatal diagnosis, assessment of viability, and subsequent management of conjoined twins.

It was not until the 18th century that plausible scientific theories began to develop as to the etiology of conjoined twins, a debate that, to some extent, continues in contemporary literature. In the 19th and early 20th centuries, conjoined twins who survived to adulthood were typically considered public attractions rather than a medical phenomenon. The most famous example is the xiphopagus twins, Chang and Eng Bunker, from whom the now obsolete term "Siamese Twins" was coined. While the Bunkers remained conjoined until their deaths at the age of 66, early attempts at separation had been made by this time, with expectedly limited success [4].

Throughout the 20th century, significant surgical milestones were achieved, with a notable breakthrough being the first successful separation of craniopagus twins in 1955. Despite these advances, separation surgery remains, in most cases, an extremely risky procedure with a serious risk of death or life-changing injuries to one or both twins [5]. In light of studies that have shown the quality of life of surviving twins who have remained conjoined to be better than expected, this raises questions over the ethical legitimacy of some separation procedures. In spite of these attempts at surgical management, the overall survivability of conjoined twins remains distressingly low, with more than 80% of conjoined twins being miscarried, stillborn, or dying perinatally [6]. Shared vital organs, commonly the liver and heart and often congenitally malformed, exclude the possibility of separation and indicate a poor prognosis.

## Conclusions

Despite the persistent fascination with conjoined twins throughout history, the rarity of the phenomenon means that they remain a poorly understood congenital anomaly. The lack of antenatal and surgical expertise in their care, coupled with the complexity and variation of their anatomy, dictates that the prognosis remains poor. It underscores the importance of conducting thorough and early antenatal visualization studies of all monozygotic twins to exclude the possibility of any anatomical fusion. Such assessment is crucial in guiding the subsequent management of those rare cases where conjoined twins are diagnosed.
